# Comparative Analysis of Context-Dependent Mutagenesis Using Human and Mouse Models

**DOI:** 10.1155/2013/989410

**Published:** 2013-08-22

**Authors:** Sofya A. Medvedeva, Alexander Y. Panchin, Andrey V. Alexeevski, Sergey A. Spirin, Yuri V. Panchin

**Affiliations:** ^1^Department of Bioengineering and Bioinformatics, Moscow State University, Vorbyevy Gory 1-73, Moscow 119992, Russia; ^2^Institute for Information Transmission Problems, Russian Academy of Sciences, Bolshoi Karetny Pereulok 19-1, Moscow 127994, Russia; ^3^Department of Mathematical Methods in Biology, Belozersky Institute, Moscow State University, Vorbyevy Gory 1-40, Moscow 119991, Russia; ^4^Department of Mathematics, Scientific Research Institute for System Studies, Russian Academy of Sciences, Nakhimovskii Prospekt 36-1, Moscow 117218, Russia

## Abstract

Substitution rates strongly depend on their nucleotide context. One of the most studied examples is the excess of C > T mutations in the CG context in various groups of organisms, including vertebrates. Studies on the molecular mechanisms underlying this mutation regularity have provided insights into evolution, mutagenesis, and cancer development. Recently several other hypermutable motifs were identified in the human genome. There is an increased frequency of T > C mutations in the second position of the words ATTG and ATAG and an increased frequency of A > C mutations in the first position of the word ACAA. For a better understanding of evolution, it is of interest whether these mutation regularities are human specific or present in other vertebrates, as their presence might affect the validity of currently used substitution models and molecular clocks. A comprehensive analysis of mutagenesis in 4 bp mutation contexts requires a vast amount of mutation data. Such data may be derived from the comparisons of individual genomes or from single nucleotide polymorphism (SNP) databases. Using this approach, we performed a systematical comparison of mutation regularities within 2–4 bp contexts in *Mus musculus* and *Homo sapiens* and uncovered that even closely related organisms may have notable differences in context-dependent mutation regularities.

## 1. Introduction

Estimates of the average point mutation rates in eukaryotic genomes usually vary between 10^−7^ and 10^−10^ mutations per nucleotide per generation [1, 2]. However, mutation rates may be dramatically altered by their genomic context. For example, there is an increased frequency of C > T mutations in the word CG in humans (and other vertebrates). This is currently attributed to the methylation of cytosines by context specific DNA methyltransferases [3]. Many other examples of context-related factors that affect mutation rates have been reported and reviewed [4–8]. Substitution rates are known to be affected by local G + C content [9], CpG density [10], recombination rates [11], proximity to small insertions or deletions [12], distance from the centromeres or telomeres [13], and the chromosome itself (e.g., the human Y chromosome has higher divergence rates than autosomes) [14]. Some of these factors might be related to each other. The study of context-dependent changes in mutation frequencies may shed light on the molecular mechanisms involved in mutagenesis [15]. Also, it is important to understand how context affects mutation rates when working in the field of molecular phylogenetics. For example, accounting for the hypermutability of certain motifs may improve the accuracy of our estimates of the divergence time between two homologous sequences [16].

Recently, it was reported that there is an increased rate of T > C mutations in the second position of the words ATTG and ATAG and an increased rate of A > C mutations in the first position of the word ACAA in the *Homo sapiens* genome [17]. This result was achieved by calculating the values called “minimal contrast” and “mutation bias” for 2–4 bp mutation contexts to evaluate if the addition of specific nucleotides to the 5′ or 3′ end of 1–3 bp words increases the probability of observing certain mutations in fixed positions. Mutation bias indicates the total excess (or deficiency) of mutations within a given mutation context. Minimal contrast indicates the excess (or deficiency) of mutations within a given context that cannot be explained by the excess (or deficiency) of mutations in one of its subcontexts.

The analysis of mutation rates for 4 bp contexts analysis requires large amounts of mutation data (millions of inferred mutations) to provide statistically significant and biologically meaningful results. Sufficient SNP data for the analysis of context-dependent mutagenesis in *H. sapiens* was available for a long time. More recently multiple whole genome sequences of *Mus musculus* were presented [18, 19]. The comparison of these genomes provides essential data on genetic divergence and context-dependent variance between mouse genetic sequences similar to that provided by human SNP analysis. We used a systematical comparison of mutation regularities within 2–4 bp contexts in *M. musculus* and *H. sapiens*, evaluated by calculating mutation bias and minimal contrasts for the contexts and uncovered a number of notable differences in context-dependent mutation regularities. Namely, we found that the aforementioned hypermutable human mutation contexts except for the excess of C > T mutations in the CG context are not hypermutable in *M. musculus*. Also, several mutation contexts are hypermutable in *M. musculus* but not in *H. sapiens*.

## 2. Methods

### 2.1. Mutation Data

We used SNP data from 17 strains of mice, available from [18] http://www.sanger.ac.uk/resources/mouse/genomes/. To reduce the possible effects of selection on protein-coding genes, we excluded SNPs present within 1000 bp of known mouse genes (UCSC genes, as in UCSC genome browser [20]). SNPs with low-coverage sequencing, near simple repeats or indels, were excluded, according to [21].

We reconstructed the ancestral states of SNPs by using the genome of SPRET/EiJ mouse as an outgroup. This is justified because this strain is the most divergent from the rest [21]. We determined the direction of mutations that happened in the remaining 16 mouse strains by comparing the observed alleles with the corresponding outgroup sequence. Only those cases were considered, when two genetic variants were present in the 16 mouse strains and one of them was present in the SPRET/EiJ strain. Further analysis was done as in [17]. A total of 12.8 million mouse SNPS were included in the analysis.

### 2.2. Mutation Context and Subcontext

We denote the mutation context of mutation *mut* in position *pos* of the word *W* as {*mut* | *pos*, *W*}. For example, {C > T | 1, CG} represents a C > T mutation in the first position of the word CG. Mutation context {*mut* | *pos*′, *W*′} is called a subcontext of the context {*mut* | *pos*, *W*} if *W*′ is a subword of *W* and any mutation *mut* occurring in position *pos* of the word *W* is at the same time a mutation occurring in position *pos*′ of the word *W*′. For example, {C > T | 1, CG} is a subcontext of {C > T | 2, ACG}. We do not study discontiguous contexts.

### 2.3. Contrast

For each pair of context {*mut* | *pos*, *W*} and its subcontext {*mut* | *pos*′, *W*′}, the value of contrast is given by the formula

(1) Contrast({mut ∣ pos,W},{mut ∣ pos′,W′}) =P{mut ∣ pos,W}P{mut ∣ pos′,W′}.

Here, *P*{*mut* | *pos*, *W*} and *P*{*mut* | *pos*′, *W*′} are the conditional probabilities of observing mutation *mut* in the position *pos* of the word *W* and in the position *pos*′ of word *W*′, respectively. Although these probabilities cannot be explicitly calculated without assumptions of the general probability of mutation per nucleotide in the genome, their ratio can be estimated by the following formula:

(2) $$\begin{array}{c} \frac{P\left\{mut\;|\;pos,W\right\}}{P\left\{mut\;|\;pos^{\prime },W^{\prime }\right\}}=\frac{N\left\{mut\;|\;pos,W\right\}/{\text{P}}_{W}}{N\left\{mut\;|\;pos,W^{\prime }\right\}/P_{W^{\prime }}}. \end{array}$$

Here, *P*_*W*_ and *P*_*W*′_ are the observed frequencies of words *W* and *W*′, respectively, among all words of the same length.

The ratio *P*_*W*_/*P*_*W*′_ estimates the probability for *W*′ to be extended to *W*. This ratio coincides with the expected ratio *N*{*mut* | *pos*, *W*}/*N*{*mut* | *pos*′, *W*′} under the hypothesis that mutations rates are the same in the context {*mut* | *pos*, *W*} and its subcontext {*mut* | *pos*′, *W*′}. Therefore, if Contrast ({*mut* | *pos*, *W*}, {*mut* | *pos*′, *W*′}) is greater than 1, it indicates an increased mutation rate in the context {*mut* | *pos*, *W*} compared with the subcontext {*mut* | *pos*′, *W*′}. Analogously, if Contrast({*mut* | *pos*, *W*}, {*mut* | *pos*′, *W*′}) is less than 1, it indicates a decreased mutation rate.

### 2.4. Minimal Contrast

For a given context {*mut* | *pos*, *W*}, let us consider all of its subcontexts {*mut* | *pos*′, *W*′}. The minimal contrast is the value *MC* = Contrast({*mut* | *pos*, *W*}, {*mut* | *pos*′, *W*′}) such that the absolute difference |*MC* − 1| is the lowest among all subcontexts {*mut* | *pos*′, *W*′}.

### 2.5. Mutation Bias

For any context {*mut* | *pos*, *W*}, there exists only one subcontext {*mut* | *pos*′, *W*′} such that the length of *W*′ is equal to 1 (i.e., *W*′ is the one-letter word, consisting of the mutated letter). The mutation bias is the contrast of the given context and this subcontext.

### 2.6. Word Frequencies

We estimated word frequencies (the fraction of a specific word in all amount of the words of the same length) in the mouse genome using [−10, −5] and [+5, +10] intervals surrounding the mouse SNPs included in our study. We used the reference mouse genome sequence for this purpose. These word frequencies were used in our calculations of mutation bias and minimal contrast for mutation contexts in *M. musculus.*

### 2.7. Statistical Significance

For a given pair of context and subcontext, let *P* = *P*_*W*_/*P*_*W*′_ be the expected probability of success in a Bernoulli trial, with the number of trials *N* = *N*{*mut* | *pos*′, *W*′} and the number of successes *K* = *N*{*mut* | *pos*, *W*}. We assume that the mutation rate for context {*mut* | *pos*, *W*} is significantly different from the mutation rate of its subcontext {*mut* | *pos*′, *W*′} if the probability to observe *K* or a more extreme number of successes out of *N* trials with the probability of success *P* is lower than a predetermined significance level. Due to large sample sizes, all obtained *P* values for context/subcontext comparisons are highly significant (*P* < 10^−15^) for all observations mentioned in our study. This remains true after correcting for multiple comparisons using the Bonferroni correction. For example, there are 1293 observed mutations for the *M. musculus* context {G > C | 3, TCGA} and 3723 mutations for its closest (with the most similar mutation bias value) subcontext {G > C | 3, TCG}. *P*_*W*_/*P*_*W*′_ for this pair is 0.081. The *P* value is much less than 10^−15^.

## 3. Results and Discussion

As shown in Table 1, among the directed mutations in *M. musculus *C > G and G > C transversions are underrepresented, compared to the fractions of such mutations among all point mutations in *H. sapiens*. Instead, C > T and G > A transitions are overrepresented in *M. musculus*. This might be due to GC-biased gene conversion being weaker in rodents [22]. Gene conversion is the transfer of genetic information between two homologous chromosomes carrying different allele variants during which one allele becomes substituted for the other. It has been shown that in mammals this process is biased in the direction that increases GC content [23]. If during recombination an *S*-*W* (where *S* is a C or G nucleotide and *W* is an A or T nucleotide) mismatched pair forms between two homologous DNA strands, the more probable scenario is that *W* will be converted into *S*. If gene conversion becomes weaker or less biased, then C > T and G > A transitions should become more frequent in observations. This is consistent with the observations of both a decrease in GC content of GC-rich isochores and an increase in GC-poor isochores in rodents [24].

Previously several hypermutable 4 bp mutation contexts were identified in *H. sapiens* [17], as shown in Table 2. We checked if these mutation regularities can be found in *M. musculus*. As shown in Table 2, only the {T > C | 2, ATTG} mutation context that is hypermutable in *H. sapiens* is also somewhat hypermutable in *M. musculus* compared to its other 4 bp contexts (among all 4 bp contexts in *M. musculus* this context is the third by minimal contrast values). However, even for this context, the observed values of mutation bias and minimal contrast are much lower than those in *H. sapiens*, indicating that context-dependent mutation regularities are very different between *H. sapiens* and *M. musculus* even at the 4 bp scale. One of our reviewers made an interesting observation that the reverse-complement image of the highly mutable *M. musculus* context {T > A | 3, TTTA} is {A > T | 2, TAAA} which is the reverse context for another highly mutable *M. musculus* context {T > A | 2, TTAA} (see Table 2). We checked if other highly mutable contexts have highly mutable reverse contexts, but this does not seem to be a general trend. Minimal contrast and mutation bias values for reverse contexts are also provided in Table 2.

We would like to explain why we make emphasis on minimal contrast and not on mutation bias, when presenting Table 2. If we sort contexts by mutation, bias all the highest ranking contexts in both *H. sapiens* and *M. musculus* will be 4 bp contexts containing the {C > G | 1, CG} context. However, most of the increase in their mutation rates is explained by the high mutation bias of the {C > G | 1, CG} context itself. Among multiple 3-4 bp contexts containing the {C > G | 1, CG} context some will inevitably have higher mutation bias than {C > G | 1, CG}, and some will have a lower mutation bias, but as long as the difference is small, these contexts are unlikely to provide interesting information about mutation regularities. Thus, we believe that minimal contrast is more informative when searching for biologically meaningful contexts.

**Figure 1:**
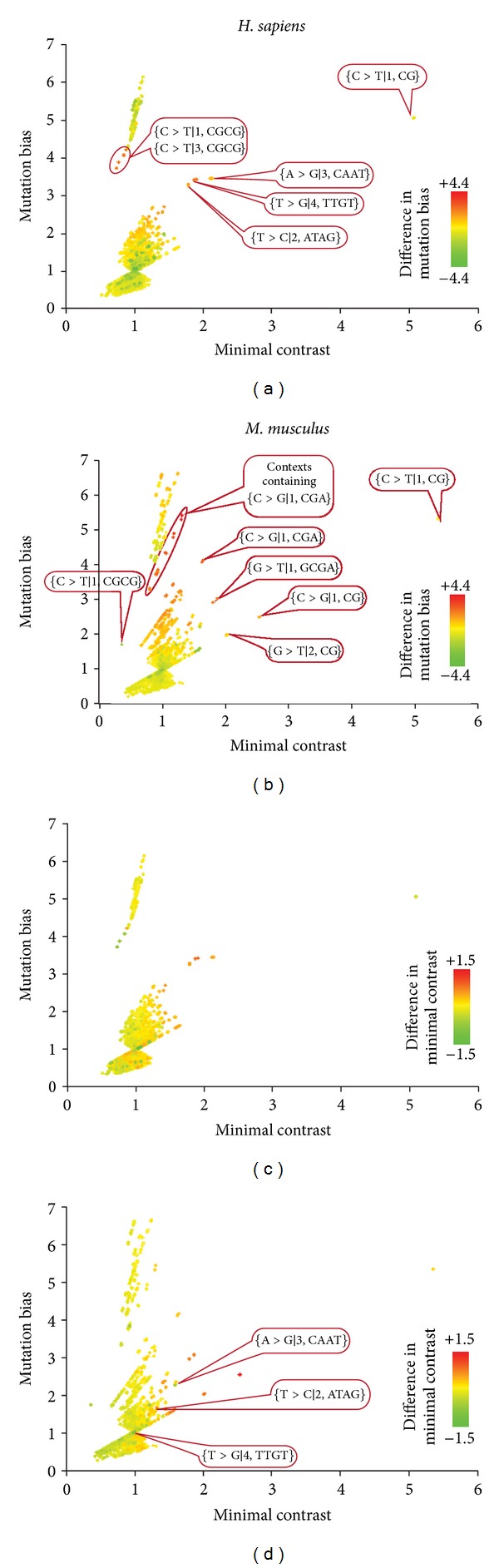
Comparison of mutation bias and minimal contrasts for all 2–4 bp mutations contexts in *H. sapiens* and *M. musculus*. Each dot represents a mutation context. The *x*-axis of each plot represents the contexts minimal contrast values, and the *y*-axis represents the contexts mutation bias. The values of mutation bias and minimal contrast are given for *H. sapiens* (plots (a) and (c)) or *M. musculus* (plots (b) and (d)). The color scheme indicates the difference between mutation biases (plots (a) and (b)) and minimal contrasts (plots (c) and (d)). Thus red dots on (a) and (c) represent contexts that are hypermutable in *H. sapiens* compared to *M. musculus*, while green dots represent contexts that are hypermutable in *M. musculus* compared to *H. sapiens*. This color scheme is reversed for (b) and (d). Note that many dots are situated in pairs; this is because complimentary mutation contexts have very similar mutation bias and minimal contrast values.

A more detailed analysis of mutation regularities is presented, in Figure 1. Previously we found it helpful to plot mutation bias versus minimal contrast for 2–4 bp contexts to identify mutation regularities with large effects. Context-dependent mutation regularities are very different between *H. sapiens* and *M. musculus*. While both species share the mutation regularity of increased C > T mutation frequency in the CG word, three hypermutable 4 bp contexts previously identified in *H. sapiens* (Figure 1(a)) are not strikingly hypermutable in *M. musculus* (Figure 1(d)). In *M. musculus* comparing to *H. sapiens*, there is also a notable increase of both mutation bias and minimal contrast values for C > G mutations in the first position of the word CGA and in contexts that include this context as a subcontext; G > T mutations in the first position of the word GCGA; C > G and G > T mutations in CG dinucleotides (Figure 1(b)). These differences in mutation patterns might reflect differences in biological mechanisms involved in primate and rodent mutagenesis.

## 4. Conclusions

We have found a number of substantial differences in context-dependent mutation regularities of *Mus musculus* and *Homo sapiens*. These differences include the reduced mutation bias and minimal contrasts for mutation contexts {T > C | 2, ATTG}, {A > C | 1, ACAA}, and {T > C | 2, ATAG} in *M. musculus* when compared to *H. sapiens*. These mutation contexts are hypermutable in *H. sapiens*. Only {T > C | 2, ATTG} is hypermutable in *M. Musculus*, but to a smaller extent than in *H. sapiens*. Mutation bias and minimal contrasts are instead increased for {C > G | 1, CGA}, {C > G | 1, CG}, {G > T | 2, CG}, and {G > T | 1, GCGA} mutation contexts in *M. musculus* when compared to *H. sapiens*.

## Figures and Tables

**Table 1:** The fractions of basic types of directed mutations, inferred from SNP data.

Mutation	Fraction	
	*H. sapiens *	*M. musculus *
A > T	0.031	0.034
T > A	0.031	0.034
A > C	0.037	0.029
T > G	0.038	0.029
C > G	0.051	0.035
G > C	0.051	0.035
G > T	0.058	0.059
C > A	0.058	0.059
T > C	0.118	0.097
A > G	0.118	0.097
C > T	0.204	0.247
G > A	0.204	0.247
Transversions	0.355	0.312
Transitions	0.645	0.688

**Table 2:** Top 5 40 bp mutation contexts by minimal contrast in *H. sapiens* and *M. musculus*. The provided subcontext is the context with the most similar to the contexts mutation bias value and is the one used for the minimal contrast calculation. Also reverse contexts are provided (contexts with the reverse mutation) with their minimal contrast and mutation bias values.

Context *{mut∣pos, W*}	Minimal contrast	Mutation bias	Subcontext *{mut∣pos′, W′}*	Reverse context	Minimal contrast	Mutation bias
*H. sapiens *						
{T > C∣2, ATTG}	2.12	3.46	{T > C∣1, TTG}	{C > T∣2, ACTG}	0.86	0.75
{A > C∣1, ACAA}	1.89	3.43	{A > C∣1, ACA}	{C > A∣1, CCAA}	1.01	1.20
{T > C∣2, ATAG}	1.78	3.29	{T > C∣2, ATA}	{C > T∣2, ACAG}	0.95	0.81
{G > C∣3, TCGA}	1.43	1.98	{G > C∣3, TCG}	{C > G∣3, TCCA}	0.59	0.37
{T > G∣4, CGGT}	1.42	2.64	{T > G∣3, GGT}	{G > T∣4, CGGG}	0.84	0.84

*M. musculus *						
{G > T∣1, GCGA}	1.83	3.00	{G > T∣1, GCG}	{T > G∣1, TCGA}	1.19	1.19
{T > A∣3, TTTA}	1.60	2.31	{T > A∣2, TTA}	{A > T∣3, TTAA}	1.47	2.55
{T > C∣2, ATTG}	1.59	2.25	{T > C∣2, AT}	{C > T∣2, ACTG}	1.01	1.01
{G > A∣4, CGCG}	1.54	1.54	{G > A∣1, G}	{A > G∣4, CGCA}	0.68	0.68
{T > A∣2, TTAA}	1.47	2.55	{T > A∣1, TAA}	{A > T∣2, TAAA}	1.60	2.31

## References

[1] Baer CF, Miyamoto MM, Denver DR (2007). Mutation rate variation in multicellular eukaryotes: causes and consequences. *Nature Reviews Genetics*.

[2] Kong A, Frigge ML, Masson G (2012). Rate of *de novo* mutations and the importance of father’s age to disease risk. *Nature*.

[3] Cooper DN, Krawczak M (1989). Cytosine methylation and the fate of CpG dinucleotides in vertebrates genomes. *Human Genetics*.

[4] Arnheim N, Calabrese P (2009). Understanding what determines the frequency and pattern of human germline mutations. *Nature Reviews Genetics*.

[5] Hodgkinson A, Ladoukakis E, Eyre-Walker A (2009). Cryptic variation in the human mutation rate. *PLoS Biology*.

[6] Blake RD, Hess ST, Nicholson-Tuell J (1992). The influence of nearest neighbors on the rate and pattern of spontaneous point mutations. *Journal of Molecular Evolution*.

[7] Hwang DG, Green P (2004). Bayesian Markov chain Monte Carlo sequence analysis reveals varying neutral substitution patterns in mammalian evolution. *Proceedings of the National Academy of Sciences of the United States of America*.

[8] Singh ND, Arndt PF, Clark AG, Aquadro CF (2009). Strong evidence for lineage and sequence specificity of substitution rates and patterns in Drosophila. *Molecular Biology and Evolution*.

[9] Smith NGC, Webster MT, Ellegren H (2002). Deterministic mutation rate variation in the human genome. *Genome Research*.

[10] Walser JC, Ponger L, Furano AV (2008). CpG dinucleotides and the mutation rate of non-CpG DNA. *Genome Research*.

[11] Lercher MJ, Hurst LD (2002). Human SNP variability and mutation rate are higher in regions of high recombination. *Trends in Genetics*.

[12] Tian D, Wang Q, Zhang P (2008). Single-nucleotide mutation rate increases close to insertions/deletions in eukaryotes. *Nature*.

[13] Hellmann I, Prüfer K, Ji H, Zody MC, Pääbo S, Ptak SE (2005). Why do human diversity levels vary at a megabase scale?. *Genome Research*.

[14] Makova KD, Li WH (2002). Strong male-driven evolution of DNA sequences in humans and apes. *Nature*.

[15] Rogozin IB, Malyarchuk BA, Pavlov YI, Milanesi L From context-dependence of mutations to molecular mechanisms of mutagenesis.

[16] Falconnet M, Behrens S (2012). Accurate estimations of evolutionary times in the context of strong CpG hypermutability. *Journal of Computational Biology*.

[17] Panchin AY, Mitrofanov SI, Alexeevski AV, Spirin SA, Panchin YV (2011). New words in human mutagenesis. *BMC Bioinformatics*.

[18] Yalcin B, Adams DJ, Flint J, Keane TM (2012). Next-generation sequencing of experimental mouse strains. *Mammalian Genome*.

[19] Wong K, Bumpstead S, van der Weyden L (2012). Sequencing and characterization of the FVB/NJ mouse genome. *Genome Biology*.

[20] Kent WJ, Sugnet CW, Furey TS (2002). The human genome browser at UCSC. *Genome Research*.

[21] Keane TM, Goodstadt L, Danecek P (2011). Mouse genomic variation and its effect on phenotypes and gene regulation. *Nature*.

[22] Clément Y, Arndt PF (2011). Substitution patterns are under different influences in primates and rodents. *Genome Biology and Evolution*.

[23] Galtier N, Piganeau G, Mouchiroud D, Duret L (2001). GC-content evolution in mammalian genomes: the biased gene conversion hypothesis. *Genetics*.

[24] Duret L, Semon M, Piganeau G, Mouchiroud D, Galtier N (2002). Vanishing GC-rich isochores in mammalian genomes. *Genetics*.

