# Corrigendum to “Comparative Analysis of Context-Dependent Mutagenesis Using Human and Mouse Models”

**DOI:** 10.1155/2020/4657615

**Published:** 2020-07-20

**Authors:** Sofya A. Medvedeva, Alexander Y. Panchin, Andrei V. Alexeevski, Sergey A. Spirin, Yuri V. Panchin

**Affiliations:** ^1^Moscow State University, Department of Bioengineering and Bioinformatics, Vorbyevy Gory 1-73, Moscow 119992, Russia; ^2^Institute for Information Transmission Problems, Russian Academy of Sciences, Bolshoi Karetny Pereulok 19-1, Moscow 127994, Russia; ^3^Department of Mathematical Methods in Biology, Belozersky Institute, Moscow State University, Vorbyevy Gory 1-40, Moscow 119991, Russia; ^4^Department of Mathematics, Scientific Research Institute for System Studies, Russian Academy of Sciences, Nakhimovskii Prospekt 36-1, Moscow 117218, Russia

As identified by a reader of the article, “Comparative Analysis of Context-Dependent Mutagenesis Using Human and Mouse Models” [1] has close similarities to another article by our group titled “Comparative Analysis of Context-Dependent Mutagenesis in Humans and Fruit Flies” [2] published in *International Journal of Genomics*, which was not cited or discussed since it was not yet published.

Previously, our group discovered several hypermutable mutation contexts in the human genome. This was published in our article “New Words in Human Mutagenesis” [3], which is cited by both articles. The goal of article [1] was to see if similar patterns can be observed in a species that is rather closely related to humans. Therefore, the mouse model was selected. For the analysis, we used SNP data from 17 strains of mice, reconstructed their ancestral states, and estimated values called “mutation bias” and “minimal contrast.” In this study, we found that “even closely related organisms may have notable differences in context-dependent mutation regularities,” described the regularities found in mice, and compared those to preexisting data on human mutagenesis.

The novel findings of article [2] were based on an entirely different dataset: 37 individual *Drosophila melanogaster* genomes were used, and *D. sechellia* and *D. erecta* genomic sequences were used as outgroups to reconstruct the ancestral states for the variable positions. Unlike mice, Drosophila are phylogenetically distant from humans and were already known to lack the most well-studied and prominent mutation regularity found in humans (and other vertebrates): an excess of C>T mutations in the CpG context. Therefore, it was interesting to compare other mutation regularities between humans and *Drosophila*, which we did thoroughly.

Both articles [1, 2] rely on strict mathematical definitions of values called “Contrast,” “Minimal Contrast,” and “Mutation bias.” For this reason, the method sections that describe how these values are calculated have high textual similarity. Both articles [1, 2] are further explorations of our previous findings about the regularities of human mutagenesis [3]; both [1, 2] explain the findings of the preceding article [3] using similar words, and thus, there is some textual similarity in the introduction sections and in the introductory part of the abstracts in [1, 2].

We apologize for not informing the editor of this journal about the existence of another article on the topic of context-dependent mutagenesis and the textual overlap. However, we would like to stress that [1, 2] are different articles, using different model organisms, different datasets, with different results (including all tables and figures) and different conclusions.

## References

[1] Medvedeva S. A., Panchin A. Y., Alexeevski A. V., Spirin S. A., Panchin Y. V. (2013). Comparative analysis of context-dependent mutagenesis using human and mouse models. *BioMed Research International*.

[2] Medvedeva S. A., Panchin A. Y., Alexeevski A. V., Spirin S. A., Panchin Y. V. (2013). Comparative analysis of context-dependent mutagenesis in humans and fruit flies. *International Journal of Genomics*.

[3] Panchin A. Y., Mitrofanov S. I., Alexeevski A. V., Spirin S. A., Panchin Y. V. (2011). New words in human mutagenesis. *BMC Bioinformatics*.

